# Parasite-host ecology: the limited impacts of an intimate enemy on host microbiomes

**DOI:** 10.1186/s42523-020-00061-5

**Published:** 2020-11-16

**Authors:** Cody S. Clements, Andrew S. Burns, Frank J. Stewart, Mark E. Hay

**Affiliations:** 1 Aquatic Chemical Ecology Center, and Center for Microbial Dynamics and Infection, School of Biological Sciences, Georgia Institute of Technology, 950 Atlantic Drive, Atlanta, GA 30332-0230 USA; 2 NIAID Microbiome Program, National Institute of Allergy and Infectious Disease, National Institutes of Health, Bethesda, MD 20892 USA; 3 Department of Microbiology & Immunology, Montana State University, Bozeman, MT 59717-3520 USA

**Keywords:** Coral reefs, *Coralliophila*, Corallivore, Gastropod, Microbial interactions, Parasite-host interactions, Parasitism

## Abstract

**Background:**

Impacts of biotic stressors, such as consumers, on coral microbiomes have gained attention as corals decline worldwide. Corallivore feeding can alter coral microbiomes in ways that contribute to dysbiosis, but feeding strategies are diverse – complicating generalizations about the nature of consumer impacts on coral microbiomes.

**Results:**

In field experiments, feeding by *Coralliophila violacea*, a parasitic snail that suppresses coral growth, altered the microbiome of its host, *Porites cylindrica,* but these impacts were spatially constrained. Alterations in microbial community composition and variability were largely restricted to snail feeding scars; basal or distal areas ~ 1.5 cm or 6–8 cm away, respectively, were largely unaltered. Feeding scars were enriched in taxa common to stressed corals (e.g. Flavobacteriaceae, Rhodobacteraceae) and depauperate in putative beneficial symbionts (e.g. Endozoicomonadaceae) compared to locations that lacked feeding.

**Conclusions:**

Previous studies that assessed consumer impacts on coral microbiomes suggested that feeding disrupts microbial communities, potentially leading to dysbiosis, but those studies involved mobile corallivores that move across and among numerous individual hosts. Sedentary parasites like *C. violacea* that spend long intervals with individual hosts and are dependent on hosts for food and shelter may minimize damage to host microbiomes to assure continued host health and thus exploitation. More mobile consumers that forage across numerous hosts should not experience these constraints. Thus, stability or disruption of microbiomes on attacked corals may vary based on the foraging strategy of coral consumers.

**Supplementary information:**

**Supplementary information** accompanies this paper at 10.1186/s42523-020-00061-5.

## Background

Tropical coral reefs are among Earth’s most biodiverse and productive ecosystems, but corals are in precipitous global decline due to a variety of physical and biotic stressors [1, 2]. The magnitude and spatial scale of these stressors are diverse – ranging from ocean-scale impacts of global change to interactions between individual corals and their associated microbes [3]. The latter have gained considerable interest, because microbial associates play both positive (e.g. nutrient uptake, pathogen resistance) and negative (e.g. disease) roles in coral health [4, 5]. Additionally, stressor-induced changes to microbiomes have been implicated in losses of beneficial, or increases of harmful, microbes (i.e. dysbiosis) [6] and often coincide with signs of coral disease [3]. Whether, and how, many of these stressors impact coral microbiomes remains unknown [7]. Filling these knowledge gaps may improve coral management and conservation in a changing ocean.

Although numerous coral stressors are due to human activities, others, such as corallivory, result from natural interactions that are common and integral to reef function and structure. Increasing evidence suggests that feeding by numerous corallivores (e.g. fishes, echinoderms, mollusks) may alter coral microbiomes in ways that contribute to dysbiosis and disease [8]. However, corallivores encompass a diverse array of organisms that vary in their feeding strategies and impacts on corals – complicating generalizations about their microbial impacts. For example, most corallivores are treated as de-facto predators, but corallivory ranges from predators that move among and kill numerous prey to parasites that associate with a single host individual for a long period and seldom kill the host [9]. These interactions share conceptual similarities (e.g. consumer-prey interactions [10];), but parasites may cause only modest suppression of host fitness, while mobile predators may kill one or more coral colonies. These differences could have important implications for understanding and predicting the effects of a given corallivore on traits that are important to coral fitness, such as susceptibility to microbial dysbiosis. As an example, mobile predators select individual prey based primarily on food quality. In contrast, sedentary parasites must select prey based not only on their value as foods, but also their suitability as habitats that provide mating sites, refuge from predators, etc. [11].

Feeding by a number of snail species negatively impacts corals [9, 12, 13], and, when in large densities, snail feeding can decimate large areas of reef (e.g. *Drupella* spp., [13]); *Coralliophila* spp., [14]). In addition to consumption, several species also suppress coral health via feeding-induced alterations to microbiomes [15, 16] or vectoring of coral disease [17, 18]. However, most studies investigating links between snail feeding and changes to coral microbiomes are correlative [19–21], and the relevant mechanisms leading to diseased states (e.g. introduction vs. enrichment of harmful microbes) remain unclear [22]. The few studies that have directly investigated changes to coral microbiomes involved generalist snails such as *Drupella* spp. [15] and *Coralliophila abbreviata* [16], both of which are mobile grazers that move among colonies removing considerable amounts of live coral tissue [23, 24]. It is unclear if corallivorous snails that are host-associated parasites produce similar impacts, despite these snails being predicted to play increasingly important roles as consumers on degraded reefs [12].

To explore these questions, we assessed how feeding by the relatively non-mobile snail *Coralliophila violacea* impacts the microbiome of its common host, *Porites cylindrica*. *C. violacea* is common on *Porites* spp. across Indo-Pacific and Red Sea reefs and is known for its “prudent” mode of feeding, which does not directly kill coral polyps or produce extensive feeding trails across colonies. Instead, the snail inserts its proboscis into the polyp’s coelenteron and slowly feeds on resources translocated from elsewhere (≥ 5 cm away) in the colony [25]. This parasitic feeding behavior allows the snail to remain stationary, producing only localized tissue damage (Fig. S1), and contributing to it previously being overlooked as a significant corallivore [12]. However, a field experiment showed that *C. violacea* feeding suppresses *P. cylindrica* growth by 18–43% depending on snail size [12]. This underscores its negative impact on *Porites* spp., which are critical foundation species and often among the few remaining corals on severely damaged reefs [26, 27]. We therefore assessed how *C. violacea* feeding altered *P. cylindrica* microbial communities – specifically testing whether microbial changes were localized to the site of feeding or were diffuse across the coral.

## Methods

We assessed the effects of *C. violacea* feeding on coral microbiomes by fragmenting and transplanting branches of *P. cylindrica* onto Namuka Reef, Viti Levu, Fiji (18°805.70′′ S, 177°23,014.94′′ E) and manipulating *C. violacea* presence and size. Briefly, 4 branches (6–8 cm in length; 18.1–55.3 g wet mass) lacking snails or snail grazing scars were fragmented from 15 separate *P. cylindrica* colonies located within a ~ 0.1 km^2^ section of fringing reef on Namuka Reef in May 2016, individually embedded within the cutoff necks of inverted plastic bottles using epoxy (Emerkit), screwed into upturned bottle caps embedded in the substrate that were spaced ~ 30–50 cm apart (total area for all outplants = ~ 90 m^2^), and individually surrounded by 1 cm^2^ grid metal screening to prevent access by other consumers (Additional file 2: Fig. S1). Following a 1-month period of acclimation and recovery from fragmentation, fragments originating from each colony were exposed to one of four treatments of *C. violacea* feeding for 24 d: either (1) no snail (control), (2) one snail ~ 8 mm in height (i.e. tip of the shell apex to the edge of the bottom lip), (3) one snail ~ 15 mm in height, or (4) one snail ~ 22 mm in height (*n* = 15 fragments per treatment). All snails began feeding within 24 h of commencing the experiment. Different sized *C. violacea* were initially used to test how snail size impacted coral growth, and we found that feeding during this 24-d period reduced *P. cylindrica* growth by 18–43%, depending on snail size [12]. Following the termination of the feeding experiment on 27 July 2016, snails were removed, and corals were immediately sampled for microbial analyses. For corals that had been subjected to snail feeding, sampling involved taking clippings from three separate locations on each coral branch: (1) within the immediate feeding area (hereafter “scar”), (2) on the opposite side of the branch ~ 1.5 cm away from where feeding had taken place (hereafter “basal”), and (3) at the top of the branch (~ 6–8 cm away from feeding, hereafter “distal”) (45 samples per snail size treatment, with 3 treatment snail sizes, for a total of 135 samples). Clippings from comparable basal and distal locations were also taken from control corals that lacked feeding snails or scars (15 samples per location, generating 30 samples total). Samples were immediately placed in WhirlPaks and stored at − 20 °C.

To assess how our outplanted corals subjected to snail feeding compared to natural colonies in the field, on 29 July 2016 we sampled individual branches from a different set of 16 haphazardly selected *P. cylindrica* colonies at Namuka Reef that were being fed upon by a single snail of ~ 15 mm in height. As with our outplants, colonies were selected from a ~ 0.1 km^2^ section of fringing reef surrounding the area where we conducted our manipulative experiment. Snails were removed, clippings were collected from scar, basal, and distal locations as described above, and quickly frozen (1 branch per colony, 3 sample types per branch, and 16 branches producing 48 total samples).

### DNA extractions and sequencing of the 16S ribosomal RNA gene

We performed Illumina sequencing of the 16S ribosomal RNA (rRNA) gene to characterize the microbial community in our samples. DNA was extracted from approximately 250 mg of coral using the Qiagen DNeasy PowerSoil Kit. For each sample, a small fragment from the original clipping (i.e. a combination of coral skeleton, tissue, and mucous) was added directly to the PowerBead tube and homogenized for 15 min by bead-beating on a Vortex-Genie 2 (Scientific Industries, Inc) with an attached 24 sample vortex adapter (Qiagen). All other steps were followed per the manufacturer’s instructions. The V3-V4 hypervariable region of the 16S rRNA gene was amplified from 1.5 μl of extracted DNA (total reaction volume of 25 μl) using Platinum PCR SuperMix (Life Technologies, Thermo Fisher Scientific, Waltham, MA) and universal 16S rRNA gene primers F515 (Parada) (5′-GTGYCAGCMGCCGCGGTAA-3′) and R806 (Apprill) (5′-GGACTACNVGGGTWTCTAAT-3′). Both primers were appended with sample-specific barcode sequences and Illumina sequencing adapters (see [28]). Primers were added to the reaction mix at a final concentration 0.2 μM, and 10 μg of bovine serum albumin (BSA, New England Biolabs Inc.) was added to help minimize effects of potential PCR inhibitors. PCR cycling conditions were: initial denaturation at 94 °C (3 min), 30 cycles of denaturation at 94 °C (45 s), primer annealing at 50 °C (45 s), primer extension at 72 °C (90 s), and final extension at 72 °C (10 min). PCR products were separated on a 1% agarose/TAE gel to verify amplicon size and lack of contamination. Products were purified using QIAquick PCR Purification Kit (Qiagen, Hilden, Germany) and purified DNA was quantified using a Qubit 2.0 fluorometer (Thermo Fisher Scientific). Equimolar concentrations of samples were pooled and sequenced on an Illumina MiSeq (Illumina Inc., San Diego, CA) using a 500-cycle kit (250 × 250 bp) with 10% PhiX to introduce sequence diversity. Illumina MiSeq sequencing resulted in a total of 40,430,368 reads with 30,479,282 reads passing the filter with a quality score > Q30. Of these reads, 5,401,800 mapped to the PhiX control genome. Of the remaining reads, ~ 77% mapped to the 16S rRNA gene with the rest of the paired-end reads mapping to chloroplast or mitochondrial sequences, potentially of host or dinoflagellate origin (Table S1).

### Microbiome data analyses

After de-multiplexing, TrimGalore! (www.bioinformatics.babraham.ac.uk/projects/trim:galore/) was used to trim low quality bases (minimum Phred score cutoff of 25), remove adaptors, and remove short sequence reads (minimum length cutoff of 100 nt). Filtered paired end sequences were then trimmed, dereplicated, merged, chimera-checked, and used to identify sequence features (exact sequence variants (ESVs)) using the DADA2 denoised-paired pipeline [29] in QIIME2 [30], with the following parameters: --p-trunc-len-f 210, −-p-trunc-len-r 190, −-p-trim-left-f 12, −-p-trim-left-r 12, −-p-max-ee-f 2, −-p-max-ee-r 2). Within the DADA2 pipeline, trimmed paired-end sequences with less than 20 nt overlap were removed. All sequence features were aligned with mafft [31] and used to construct a phylogeny with fasttree2 [32] within QIIME2. Taxonomy was assigned to features by comparison to the SILVA ribosomal RNA database (Release 132) using the q2-feature-classifier classify-sklearn naïve Bayesian taxonomy classifier. ESVs mapping to chloroplast or mitochondrial sequences were filtered from the table (Additional file 1). Diversity analyses (calculation of ESV richness, Shannon diversity) were performed in QIIME2. These analyses were done using sequences rarefied (subsampled without replacement; 157 samples total) to a uniform count of 8013 sequences per sample (rarefaction done in phyloseq using the rarefy_even_depth function; Additional file 2: Fig. S2); this count maximized the amount of samples for downstream analyses while retaining more than 90% of total diversity for the majority of samples. Rarefying microbial datasets has been shown to accurately distinguish sample groups based on microbiome composition [33] – the goal of this study – and to decrease false discovery rates in datasets with large variation in library size (~10X variation) such as ours (1369–106,282 reads per sample; Additional file 1). However, rarefaction is not universally agreed upon [34]; thus, we also conducted all analyses using non-rarefied datasets (Additional file 2: Fig. S4, S6, S7, S8, S11, & S12; 197 samples total). Findings from analyses of non-rarefied datasets were consistent with analyses of rarefied datasets.

Analyses of microbiome data were conducted in a stepwise manner to test whether and how snail feeding impacted *P. cylindrica* microbiomes. Questions, in order of analysis, were as follows:

1. *Among our outplanted corals with snails, did microbiomes of locations that lacked feeding (distal & basal) differ as a function of snail size? Did they differ from basal and distal locations on outplanted corals lacking snails (controls) or natural colonies on which intermediate sized snails were feeding?*
2. *Did microbiomes of scars on outplanted corals differ as a function of snail size, or from snail scars on natural colonies?*
3. *Among outplanted corals subjected to snail feeding, did microbiomes differ based on sampling location (scar, basal, or distal)?*
4. *Among outplanted corals lacking snail feeding, did microbiomes differ based on sampling location (basal* vs. *distal)?*
5. *Among natural colonies, did microbiomes differ based on sampling location (scar, basal, or distal)?*

In each case, Bray-Curtis dissimilarity values were calculated using the distance function in PRIMER-e [35]. Weight metrics such as Bray-Curtis dissimilarity are necessary to prevent minimizing the contribution of abundant ESVs, such as members of the Endozoicomonadaceae family that are key to coral health and may be present in all samples, but dominant only in specific ones. Principal coordinate analysis (PCO) and corresponding tests for differences in microbiome composition (permutational multivariate analysis of variance, PERMANOVA) were implemented in Primer E [35] via one-factor tests, with the parent colony of each replicate coral branch included as a random factor in all analyses. To test for differences in microbiome variability, we used the PERMDISP function in PRIMER-e to obtain dispersion values (deviation from the centroid) for all relevant analyses. Differences in dispersion among treatments (e.g. sampling locations) were tested with linear mixed effects (LME) models in R (v. 4.0.2) [36] using the package nlme [37]. The parent colony of each replicate coral branch was incorporated as a random factor into all analyses, and when necessary, the varIdent function was used to control for heteroscedasticity. If significant, subsequent multiple comparisons of means were performed using the generalized linear hypothesis test (glht) and Tukey test in the multcomp package [38]. Alpha diversity (ESV richness, Shannon diversity) of relevant datasets was calculated using the estimate_richness function in phyloseq. Differences among relevant treatments were conducted using LME models, with parent coral colony as a random factor. Subsequent multiple comparisons were conducted using glht and Tukey tests as described above. For each analysis, significance levels were Bonferroni adjusted to correct for multiple comparisons of relevant datasets.

For each analysis where we detected significant differences in microbiome composition and variance, we also developed a supervised classification model using random forest analysis (a machine learning method, QIIME2 function classify-samples with 80% of the dataset used to train the classifier and 20% used to test the model) to quantify the accuracy of predicting a particular sample’s grouping (e.g. Sample treatment in Question 2; Sample location in Questions 3&5) based on taxonomic composition (exact sequence variants, ESVs). Log_2_ fold change of ESVs between sample conditions was calculated in R (v. 3.3.2) in the DESeq2 package (v. 1.24.0) using Wald’s test with a parametric fit. The shrinkage estimator function in DESeq2 is particularly useful for count data with potentially high within-group variability, as can occur in microbiome data from experimental studies with relatively low sample numbers [39].

## Results

### Microbiomes of basal and distal locations that lacked snail feeding

When we compared basal samples across all treatments and then distal samples across all treatments (i.e. control outplants, outplants with 8 mm, 15 mm, or 22 mm snails, and natural colonies), neither contrast exhibited significant differences in microbiome diversity (LME, ⍺ = 0.025; basal, ESV richness: *p* = 0.713, Shannon diversity: *p* = 0.847; distal, ESV richness: *p* = 0.930, Shannon diversity: *p* = 0.479), composition (PERMANOVA, ⍺ = 0.025; basal, *p* = 0.176; distal, *p* = 0.282) or variability (LME, ⍺ = 0.025; basal, *p* = 0.351; distal, *p* = 0.205) (Additional file 2: Fig. S3 & S5). This was also the case for comparisons involving non-rarefied datasets (Additional file 2: Fig. S4 & S6). Thus, among outplanted corals, and in comparison to natural colonies, we were unable to detect differences in coral microbiomes at similar locations (e.g. basal locations) where snails were not directly feeding.

### Microbiomes of feeding scars

**Fig. 1:**
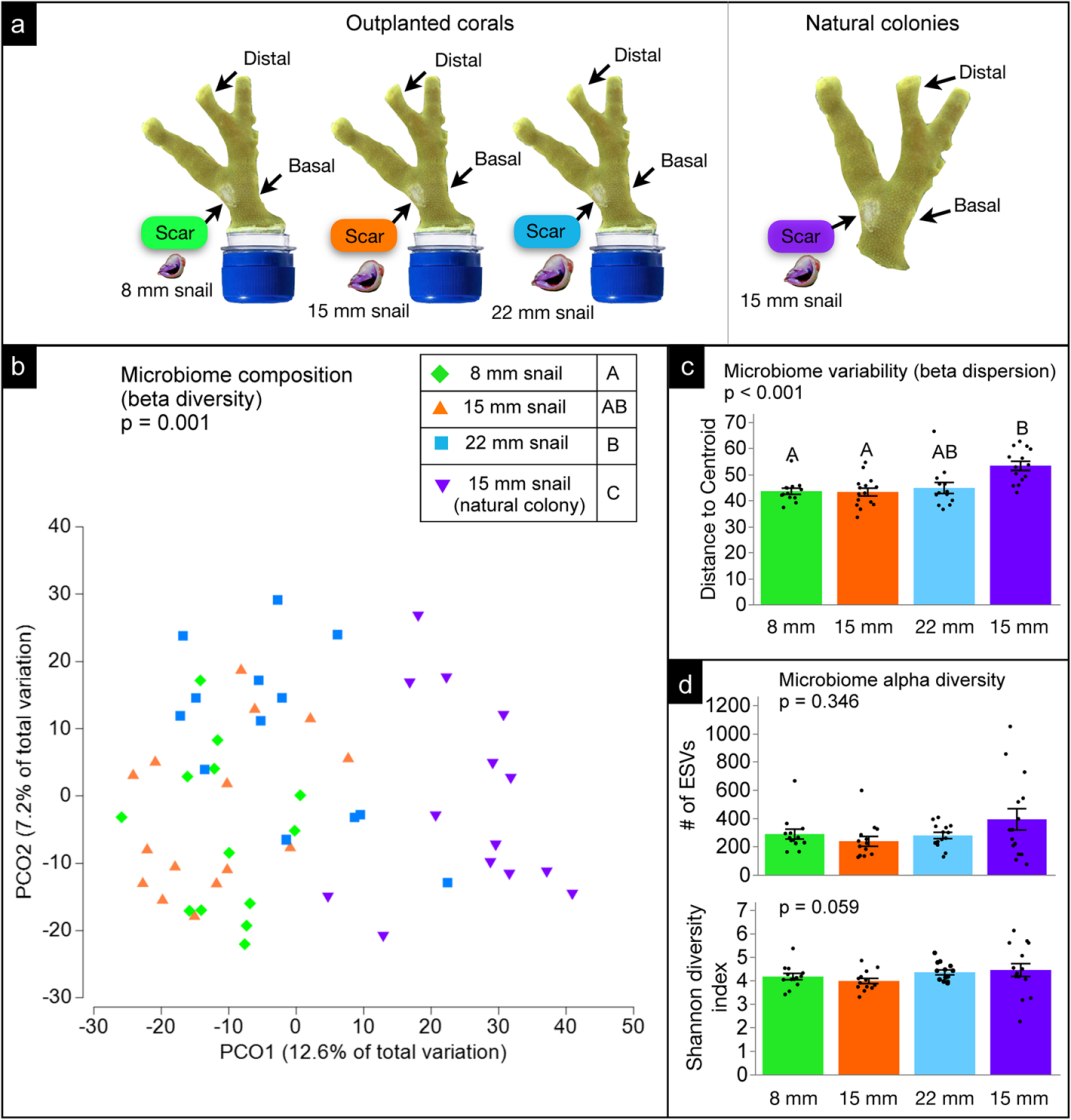
Microbiomes of feeding scars. **a** Sampling schematic of scar locations from outplanted corals in our manipulative experiment and natural colonies in the field used for analyses (one-factor test, factor: treatment). **b** Microbiome composition (beta diversity) of scar samples among treatments. Letters in legend denote significant differences (*p* < 0.01). **c** Microbiome variability (beta dispersion) of scar samples among treatments (⍺ = 0.025). **d** Microbiome alpha diversity of scar samples among treatments (⍺ = 0.025). Analyses were performed following rarefaction (subsampling without replacement) to a uniform sequence count of 8013 sequences per sample

Microbiomes of feeding scars differed significantly among treatments in composition (PERMANOVA, ⍺ = 0.025, *p* = 0.001, Fig. 1b) and variability (LME, ⍺ = 0.025, *p* < 0.001, Fig. 1c), but not diversity (LME, ⍺ = 0.025; ESV richness: *p* = 0.346; Shannon diversity: *p* = 0.059, Fig. 1d). Pairwise comparisons indicated that microbiome composition of scars on natural colonies differed significantly from scars on outplanted corals fed on by 8 mm, 15 mm, or 22 mm snails. Microbiome composition of scars on outplanted corals only differed significantly between 8 mm and 22 mm snails (⍺ = 0.01, *p* = 0.003, Fig. 1b). Microbiome variability of scars on natural colonies significantly differed from that of scars on outplants hosting 8 mm and 15 mm snails (*p* < 0.001), but not 22 mm snails (*p* < 0.014). Variability did not differ among outplanted corals as a function of snail size (⍺ = 0.01, *p* = 0.931–0.999, Fig. 1c). Analyses of non-rarefied data exhibited similar trends to rarefied data analyses – only differing in that microbiome composition of scars did not significantly differ between outplants hosting 8 mm and 22 mm snails (PERMANOVA, ⍺ = 0.025, *p* = 0.044) and microbiome variability of scars did not significantly differ among treatments (LME, ⍺ = 0.025, *p* = 0.031, Additional file 2: Fig. S7).

**Fig. 2:**
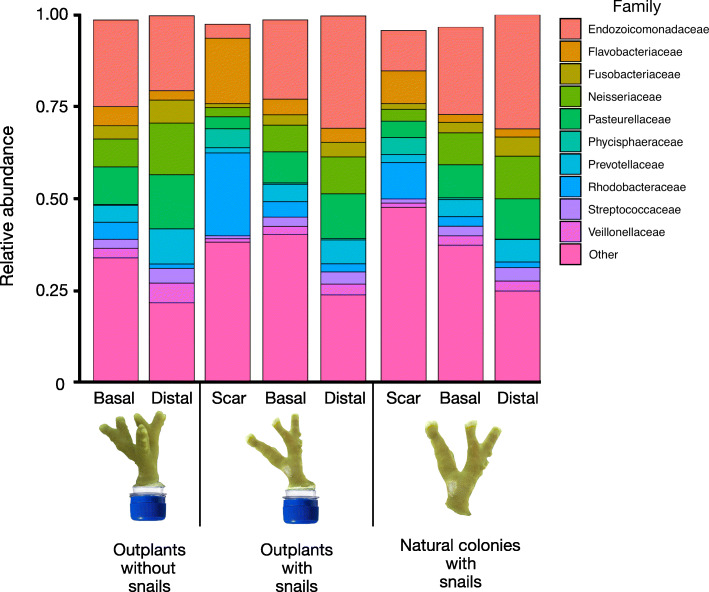
Average microbial community composition. Data rarefied to 8013 sequences per sample for *P. cylindrica* outplants without snails (left, *n* = 8 [Basal], 7 [Distal]) or with snails (center, *n* = 43 [Scar], 36 [Basal], 39 [Distal]), and natural colonies with snails (right, *n* = 15 [Scar], 13 [Basal], 12 [Distal]). Taxa are grouped by family, with the ten most abundant families depicted separately. All other families were pooled and depicted as ‘Other’

Random forest analysis indicated that samples from outplants or natural colonies could be predicted with 100% accuracy from bacterial community membership at the ESV level. There were few ESVs differentially abundant when comparing outplanted coral scars to natural scars (Additional file 3). Taxonomic groups known to be composed of strict anaerobes were enriched in scars on outplanted corals. A Ruminococcaceae ESV was undetected in scars on natural colonies but averaged 0.7% of sequences on outplanted scars. Similarly, a Clostridiaceae ESV comprised less than 0.001% of sequences on natural scars but 1.6% of sequences on outplanted scars (Fig. 2).

### Microbiomes of scar, basal, & distal locations on outplanted corals with snails

**Fig. 3:**
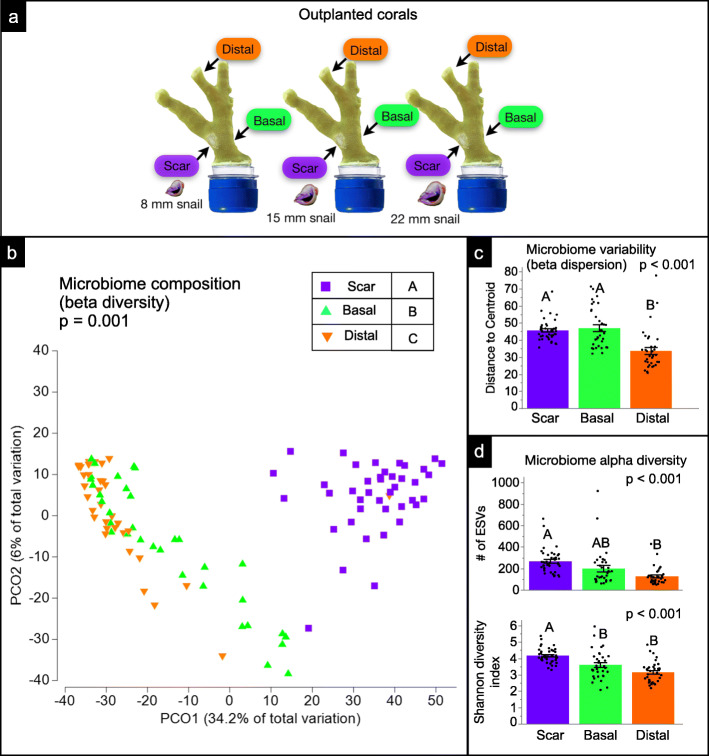
Microbiomes of scar, basal, & distal locations on outplanted corals with snails. **a** Sampling schematic of locations from corals in our manipulative experiment that were subjected to snail feeding used for analyses (one-factor test, factor: location). **b** Microbiome composition (beta diversity) of samples by location. Letters in legend denote significant differences (*p* < 0.01). **c** Microbiome variability (beta dispersion) of samples by location. Letters in legend denote significant differences (*p* < 0.01). **d** Microbiome alpha diversity of samples by location. Letters in legend denote significant differences (*p* < 0.01). Analyses were performed following rarefaction (subsampling without replacement) to a uniform sequence count of 8013 sequences per sample

When evaluating microbiomes of scar, basal, and distal locations on coral outplants that were subjected to snail feeding, we detected significant differences among locations in terms of microbiome composition (PERMANOVA, ⍺ = 0.025, *p* = 0.001, Fig. 3b), variability (LME, ⍺ = 0.025, *p* < 0.001, Fig. 3c), and diversity (LME, ⍺ = 0.025; ESV richness: *p* < 0.001, Shannon diversity: *p* < 0.001, Fig. 3d). Pairwise comparisons of locations indicated that scar, basal, and distal samples all differed significantly from one another in microbiome composition (⍺ = 0.01, *p*  0.006, Fig. 3b). Microbiome variability was statistically indistinguishable between scar and basal locations (*p* = 0.815), but variability of both of these locations was significantly greater than variability of distal locations (⍺ = 0.01, *p* < 0.001, Fig. 3c). In contrast, microbiome diversity was significantly greater for scar vs. distal locations (⍺ = 0.01, *p* < 0.001), while basal and distal did not differ significantly (⍺ = 0.01, *p* = 0.029–0.060, Fig. 3d). Significant differences between scar vs. basal locations depended on the diversity metric of interest (LME, ⍺ = 0.01, ESV richness: *p* = 0.119, Shannon diversity: *p* < 0.001, Fig. 2d). These findings were consistent with those based on a non-rarefied dataset, with the exception that scar and basal locations significantly differed regardless of the diversity metric used (LME, ⍺ = 0.01, ESV richness: *p* = 0.001, Shannon diversity: *p* < 0.001, Additional file 2: Fig. S8).

When quantifying the accuracy with which a sample’s location could be predicted from bacterial community membership at the ESV level, our overall model had an accuracy of 92% – a 2.6-fold improvement on error rates compared to random guessing – and was 100, 88, and 89% accurate at predicting scar, basal, and distal samples, respectively. Scars had a greater proportion of sequences assigned to the family Rhodobacteraceae (22.5%) compared to basal (4.2%) and distal (2.2%) locations. Flavobacteriaceae sequences were also enriched in scars (17.8%) compared to basal (4.3%) and distal (3.8%) locations. The increase in these families corresponded with a decrease in the proportion of sequences assigned to the family Endozoicomonadaceae (Fig. 2). Differential abundance analysis via DESeq2 (log2 fold change > 2, FDR-adjusted *P*-value < 0.01) indicated that multiple ESVs in both the Flavobacteriaceae and Rhodobacteraceae were enriched in scar samples. Within the Flavobacteriaceae, many of these ESVs were closely related to the genera *Spongiivirga* and *Muricauda*. In the Rhodobacteraceae, most notably an ESV related to the genus *Ruegeria* was enriched 24.6-fold and 24.7-fold (average of ~ 14% of community composition) compared to basal and distal samples (average of < 1%), respectively (Additional file 2: Fig. S9; Additional file 4). Multiple ESVs related to the genus *Phycisphaera* in the phylum Planctomycetes were also enriched (6–25-fold) within scar samples (Additional file 2: Fig. S9).

### Microbiomes of outplanted corals that lacked snail feeding (basal & distal locations)

Microbiomes of basal and distal samples on outplanted corals not subject to snail feeding (controls) did not differ significantly in composition (PERMANOVA, ⍺ = 0.025, *p* = 0.032), variability (LME, ⍺ = 0.025, *p* = 0.031, or diversity (LME, ⍺ = 0.025; ESV richness, *p* = 0.232; Shannon diversity, *p* = 0.148) or (Additional file 2: Fig. S10). This was also true when analyzed using non-rarefied data (Additional file 2: Fig. S11).

### Microbiomes of scar, basal, & distal locations on natural colonies with snail feeding

**Fig. 4:**
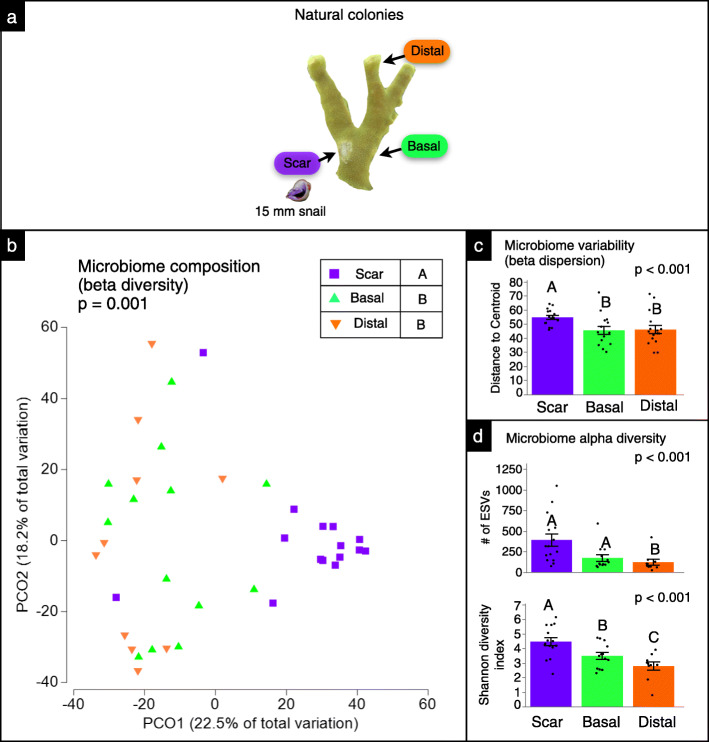
Microbiomes of scar, basal, & distal locations on natural colonies with snail feeding. **a** Sampling schematic of locations from natural coral colonies that were subjected to snail feeding used for analyses (one-factor test, factor: treatment). **b** Microbiome composition (beta diversity) of samples by location. Letters in legend denote significant differences (*p* < 0.01). **c** Microbiome variability (beta dispersion) of samples by location. **d** Microbiome alpha diversity of samples by location. Letters in legend denote significant differences (*p* < 0.01). Analyses were performed following rarefaction (subsampling without replacement) to a uniform sequence count of 8013 sequences per sample

Using the rarefied dataset, microbiomes of natural colonies being attacked by snails differed significantly among sampling locations in composition (PERMANOVA, ⍺ = 0.025, *p* = 0.001, Fig. 4b), variability (LME, ⍺ = 0.025, *p* < 0.001, Fig. 4c), and diversity (LME, ⍺ = 0.025, ESV richness, *p* < 0.001; ANOVA, ⍺ = 0.025, Shannon diversity, *p* < 0.001, Fig. 4d). Pairwise comparisons between locations indicated that both microbiome composition and variability of scar samples differed significantly from basal and distal samples (LME, ⍺ = 0.01, composition: *p* = 0.001, Fig. 4b; variability: *p* 0.004 Fig. 4c), but basal and distal samples did not differ from each other (LME, ⍺ = 0.01, composition: *p* = 0.260, Fig. 4b; variability: *p* = 0.806, Fig. 4c). Microbiome diversity of scar and basal samples significantly differed from distal samples (LME, ⍺ = 0.01, ESV richness: *p*  0.003; Shannon diversity: *p*  0.001, Fig. 4b), while significant differences between scar and basal samples depended on the diversity metric used (⍺ = 0.01, ESV richness: *p* = 0.010; Shannon diversity: *p* < 0.001, Fig. 4b). When data were not non-rarefied, microbiome composition and variability significantly differed, with scar samples differing from basal and distal samples in all cases. Microbiome diversity of scar samples significantly differed from basal and distal locations in all cases (⍺ = 0.01, ESV richness: *p* < 0.001; Shannon diversity: *p* < 0.001, Additional file 2: Fig. S12), but differences between basal and distal samples depended on the diversity metric used (ESV richness: *p* = 0.706; Shannon diversity: *p* = 0.001, Additional file 2: Fig. S12).

Random forest analysis revealed that sample locations could be predicted with 70% accuracy, based on bacterial community membership at the ESV level. The model was 75, 67, and 67% accurate at predicting scar, basal and distal samples, respectively. Similar to the outplanted corals, scar samples of natural colonies exhibited a greater proportion of sequences belonging to the Rhodobacteraceae and Flavobacteriaceae families. Rhodobacteraceae represented ~ 10% of scar sequences, but only 2.6 and 1.5% of basal and distal sequences, respectively. Flavobacteriaceae represented 8.9% of scar sequences, but only ~ 2% of both basal and distal sequences. Conversely, Endozoicomonadaceae represented 11% of scar sequences compared to 23.8 and 31% of basal and distal sequences, respectively (Fig. 2). Patterns of differential ESV abundance were also similar to those of outplanted corals. ESVs affiliated with *Mauricauda* (Flavobacteriaceae), *Ruegeria* (Rhodobacteraceae), *Rubritalea* (Rubritaleaceae), *Phycisphaera* (Planctomycetes), and *Spongiivirga* (Flavobacteriaceae) were significantly enriched in scars compared to basal and distal sites (Additional file 2: Fig. S13).

## Discussion

### *C. violacea* feeding impacts on coral microbiomes are localized

Determining the relevant scales at which ecological interactions operate is key to discerning their effects on ecosystem dynamics and processes [40]. For interactions between organisms and their natural enemies, the extent of negative impacts will depend in part on enemy traits (e.g. feeding mode, habitat use) that are integral to prey or host exploitation. We found that feeding by the parasitic corallivore *C. violacea* altered coral microbiomes in ways that may be indicative of dysbiosis. These included shifts in microbial community composition and increased community variability, as well as decreased prevalence of putative symbionts and increases in potentially harmful taxa. However, these effects were largely restricted to feeding scar locations. Like other consumers with intimate ties to their host [41–43], a parasite like *C. violacea* may benefit from limiting negative impacts to its host, upon which it depends not only for food, but also for habitat and refuge from predation [12]. In contrast, consumers that are more mobile and feed from many individuals may be less affected by the death or dysbiosis of any individual prey and thus under less selection to minimize disruption to their preys’ microbiomes [44]. That said, previous studies assessing coral microbiomes (e.g. composition, variability) in response to corallivory focused on microbiome samples that pooled areas of the coral where feeding had and had not occurred; they were not focused on localized differences among feeding scar and non-scar locations [15, 16]. Thus, it is difficult to assess whether the differences they reported were due to changes across the entire coral or changes occurring primarily at feeding sites that were averaged with lesser changes from locations without feeding. For the corallivore *C. violacea*, which remains stationary as it consumes nutrients transported from elsewhere in the colony, effects on the coral’s microbiome were relatively localized and had minimal detectable impacts at distance from the feeding scar.

### Microbiomes of non-scarred, basal and distal locations did not differ

When comparing non-scar locations among treatments, microbial communities did not differ significantly in diversity, composition, or variability. This was the case for locations i) both near (≤1.5 cm, basal) and more distant (6–8 cm, distal) from snail feeding in our outplants, ii) for natural colonies in the field, and iii) for basal versus distal locations on control outplants that lacked snails. These findings suggest that non-feeding locations on corals in our outplants were fairly representative of comparable locations on outplants without snails, as well as colonies in the field where snails were naturally present [12]. In addition, neither basal nor distal samples significantly differed among outplanted corals hosting either an 8, 15, or 22 mm snail, despite significant reductions (18–43%) in coral growth with increasing snail size. This suggests that the microbial impacts of feeding are spatially constrained, despite colony-wide effects for traits like growth.

### Feeding scar microbiomes differed between outplants vs. natural colonies

In contrast to coral locations that lacked snail feeding, we did observe significant differences in microbiome composition and variability, but not diversity, between feeding scars in our outplants vs. feeding scars on natural colonies. The reasons for these compositional differences are unknown but may be due to differences between outplants vs. natural colonies in colony size, snail abundance, or duration of feeding history. For example, ESVs of anaerobic bacteria (Ruminococcaceae, Clostridiaceae) were among the few sequences that were significantly enriched in the scars of our coral outplants, and anaerobic metabolism is often enriched at lesion sites within diseased corals as tissue decays [45, 46]. Recent (~ 24 d) scars from our outplants may have experienced greater anaerobe colonization or enrichment than older, more-established scar communities on natural colonies. Furthermore, colony size may be another reason for differences in scar microbiome composition between outplants vs. natural colonies. *C. violacea* feeds on resources that are translocated to the wound site from other parts of the coral colony [25], but the dynamics of this process may be altered for our smaller (6–8 cm) coral outplants versus the larger intact colonies. That said, it is interesting to note that, as with basal and distal samples, scar microbiomes exhibited limited differences among outplants that hosted different-sized snails, despite snails decreasing coral growth by 18–43% depending on snail size [12]. This again suggests that impacts of this snail’s feeding on coral microbiomes may be constrained relative to other factors (e.g. growth) that are influenced by snail feeding.

### Microbiomes of feedings scars were distinct from non-feeding locations

When comparing outplanted corals fed on by snails, we found that scar, basal, and distal locations all differed significantly from each other in microbiome composition (Fig. 3). These differences, including between basal and distal samples, were likely influenced by snail feeding, since basal and distal samples in controls lacking snails did not exhibit significant differences in microbiome composition, variability, or diversity (Additional file 2: Fig. S10 & S11). Moreover, microbiome variability was significantly greater in both scar and basal vs. distal samples. Elevated microbiome variability occurs in response to stress in a number of animal hosts, including corals, and is often considered indicative of dysbiosis [6]. Our findings suggest that *C. violacea* feeding may impair a coral’s ability to regulate its microbiome near the site of snail feeding and is consistent with other studies that have found increased microbiome variability following corallivory [15, 16] or mechanical wounding [47]. In this case, however, these effects are likely more localized than pervasive – operating at or near the location of feeding. Basal locations differed from distal locations in microbiome composition and variability, but not diversity, and suggest that effects of feeding on the coral’s microbiome decrease with distance from the site of feeding.

Comparisons among locations on natural colonies further support the notion that *C. violacea* feeding effects are localized. Scar microbiomes significantly differed in composition, and were more variable, when compared to basal and distal locations. However, unlike our outplants, basal and distal microbiomes did not differ in composition and variability for natural colonies in the field. Differences between findings from our outplants and natural colonies could again be due to the ratio of colony mass to snail mass and the potentially greater access to translocatable resources from the larger, intact colonies we sampled in the field. A second hypothesis is that newly initiated feeding disrupts both scar microbiomes and the microbiome of nearby tissues, but that the beyond-scar effect dissipates over time as the coral adapts to the initial feeding stress. At present, the weight of evidence suggests that the microbial impacts of feeding are spatially constrained.

### Scar microbiomes were depauperate in putative bacterial symbionts and enriched in potential bacterial opportunists

Scars of both outplants and natural colonies were depauperate in sequences from the family Endozoicomonadaceae compared to their respective basal or distal locations. Members of this family are generally considered putative symbionts and are common among healthy corals while being underrepresented among corals subjected to various stressors [3], including corallivore predation (e.g. *Drupella* snails, [15]). Increasing evidence also suggests that the Endozoicomonadaceae play key roles in regulating their coral host’s health and response to stress [3]. In contrast, scars were enriched in numerous ESVs from the Flavobacteriaceae, Rhodobacteraceae, Planctomycetes, and Verrucomicrobia when compared to their respective basal and distal locations. Various members of the families Flavobacteriaceae and Rhodobacteraceae are opportunists that colonize stressed and diseased corals [3, 48, 49], and all four bacterial groups possess diverse pathways for metabolizing various proteins and sugars. Such microbial changes might be expected for scars in which the microbes are consuming diverse organic compounds (e.g. amino acids, nucleic acids, lipids, etc.) associated with stressed or decaying tissue. Multiple ESVs closely related to the genus *Muricauda* (Flavobacteriaceae) were enriched in both scar types, and genomic analysis of a representative species showed a high percentage of genes encoding for amino acid transport and metabolism [50]. *Rubritalea,* a Verrucomicrobia genus, comprised 1.3 and 5.4% of the scar community of outplanted and natural corals, respectively. Species from this genus have been isolated from marine sponges and are capable of growing on diverse sugars and polysaccharides [51]. Multiple scar-enriched ESVs were closely related to the Planctomycetes genus *Phycisphaera*. Planctomycetes are widespread in biofilm communities of macroalgae [52], and a representative of *Phycisphaera* has the metabolic potential to grow on sugars and polysaccharides produced by its macroalgal host [53]. Multiple ESVs related to the Rhodobacteraceae were also enriched in scars. One of these ESVs, related to the genus *Ruegeria*, was particularly dominant, representing 14 and 5.3% of scar communities of outplants and wild corals, respectively. *Ruegeria* spp. typically have large genomes with highly versatile metabolic capabilities [54], and some have been shown to produce both algicidal and bactericidal compounds in culture. Indeed, cultures of coral-associated *Ruegeria* have been shown to inhibit growth of the coral pathogen *Vibrio coralliilyticus* [55]. While the vast majority of scar-associated ESVs are likely opportunists, these specific community members could be involved in limiting dysbiosis to the vicinity of the scar.

## Conclusions

Consumers, including corallivores, employ a wide variety of feeding strategies ranging from predation to parasitism – each of which is shaped by various ecological and evolutionary constraints [10]. Unlike other well-known, mobile corallivores (e.g. *Acanthaster* sea stars, parrotfishes, *Drupella* snails) that cause widespread coral damage and have been implicated in coral microbiome dysbiosis or as potential disease vectors [15–17, 56–60], *C. violacea* is a corallivorous parasite known for its sessile mode of feeding that visually results in only minor, and localized, tissue damage. Our findings suggest that this feeding strategy also results in only localized impacts to the coral microbiome, which might be expected for a corallivore that shelters on, and exploits, its host for extended periods of time. Previous studies suggested that molluscs, such as *C. violacea*, feed without moving to decrease exposure of coral skeleton that may aid predatory fish in ‘tracking’ snails [61], while others have suggested that this strategy may help avoid the need to move between hosts, a time in which snails seem especially susceptible to consumers [12]. That said, recent evidence suggests that *C. violacea* feeding scars can exhibit progressive tissue loss following snail removal – putatively due to processes associated with secondary colonization by algae [62]. Thus, there may be a selective advantage to feed in ways that limit adverse, secondary impacts to the coral host, including alterations to the composition and stability of its microbiome. Such “optimal virulence” would be consistent with theory [63, 64] and may provide useful insights for understanding and predicting the effects of sedentary consumers more broadly [10, 41]. The spatial specificity of our findings also suggests rethinking future assessments of consumer impacts on host microbiomes.

## Supplementary information

**Additional file 1.** Type (outplant vs natural colony), location (basal, distal, or scar), and sequencing read count statistics of samples used to assess microbiome composition by Illumina 16S rRNA gene sequencing. Raw counts of paired end sequences per sample (column D) are shown relative to counts after trimming with TrimGalore (column E), after paired-end read merging (column F), and after assignment to an exact sequence variant (ESV) table filtered to remove sequences matching chloroplast and mitochondrial 16S rRNA genes (column G).

**Additional file 2 Fig. S1.** (a) *Coralliophila violacea* (red arrows) feeding on natural colonies of *Porites cylindrica* in the field. (b) *C. violacea* feeding scars (red arrows) on natural *P. cylindrica* colonies. (c) Experimental set-up assessing the effects of *C. violacea* feeding on *P. cylindrica* growth and survivorship. (d) *C. violacea* feeding scars (red arrows) at the end of the 24 day experiment. **Fig. S2:** ESV rarefaction curves for each of the 76 coral samples we evaluated. The vertical line indicates sampling depth (8013 sequences). **Fig. S3:** (a) Sampling schematic of basal locations (using rarefied data) from corals in our manipulative experiment and natural colonies in the field used for analyses (one-factor test, factor: treatment). (b) Microbiome composition (beta diversity) of basal samples among treatments. (c) Microbiome variability (beta dispersion) of basal samples among treatments. (d) Microbiome alpha diversity of basal samples among treatments. Analyses were performed following rarefaction (subsampling without replacement) to a uniform sequence count of 8013 sequences per sample. **Fig. S4:** (a) Sampling schematic of basal locations (using non-rarefied data) from corals in our manipulative experiment and natural colonies in the field used for analyses (one-factor test, factor: treatment). (b) Microbiome composition (beta diversity) of basal samples among treatments. (c) Microbiome variability (beta dispersion) of basal samples among treatments. (d) Microbiome alpha diversity of basal samples among treatments. **Fig. S5:** (a) Sampling schematic of distal locations (using rarefied data) from corals in our manipulative experiment and natural colonies in the field used for analyses (one-factor test, factor: treatment). (b) Microbiome composition (beta diversity) of distal samples among treatments. (c) Microbiome variability (beta dispersion) of distal samples among treatments. (d) Microbiome alpha diversity of distal samples among treatments. Analyses were performed following rarefaction (subsampling without replacement) to a uniform sequence count of 8013 sequences per sample. **Fig. S6:** (a) Sampling schematic of distal locations (using non-rarefied data) from corals in our manipulative experiment and natural colonies in the field used for analyses (one-factor test, factor: treatment). (b) Microbiome composition (beta diversity) of distal samples among treatments. (c) Microbiome variability (beta dispersion) of distal samples among treatments. (d) Microbiome alpha diversity of distal samples among treatments. Analyses were performed using a non-rarefied dataset. **Fig. S7:** (a) Sampling schematic of scar locations (using non-rarefied data) from corals in our manipulative experiment and natural colonies in the field used for analyses (one-factor test, factor: treatment). (b) Microbiome composition (beta diversity) of distal samples among treatments. (c) Microbiome variability (beta dispersion) of distal samples among treatments. (d) Microbiome alpha diversity of distal samples among treatments. **Fig. S8:** (a) Sampling schematic of locations (using non-rarefied data) from corals in our manipulative experiment that were subjected to snail feeding used for analyses (one-factor test, factor: location). (b) Microbiome composition (beta diversity) of samples by location. Letters in legend denote significant differences (*p* < 0.01). (c) Microbiome variability (beta dispersion) of samples by location. Letters in legend denote significant differences (*p* < 0.01). (d) Microbiome alpha diversity of samples by location. Letters in legend denote significant differences (*p* < 0.01). **Fig. S9:** Heat map displaying differentially abundant (log_2_ fold > 2) for those ESVs that were greater than 0.5% of the community in either scar, basal, or distal locations on coral outplants fed on by snails. Most ESVs could be identified to the genus or family level (right). Letters indicate significant groupings (FDR-adjusted *p* < 0.01) via Wald’s test with a parametric fit. **Fig. S10:** (a) Sampling schematic of basal and distal locations (using rarefied data) from control corals in our manipulative experiment that lacked snails used for analyses (one-factor test, factor: location). (b) Microbiome composition (beta diversity) of samples by location. (c) Microbiome variability (beta dispersion) of distal samples among treatments. (d) Microbiome alpha diversity of distal samples among treatments. Analyses were performed following rarefaction (subsampling without replacement) to a uniform sequence count of 8013 sequences per sample. **Fig. S11:** (a) Sampling schematic of basal and distal locations (using non-rarefied data) from control corals in our manipulative experiment that lacked snails used for analyses (one-factor test, factor: location). (b) Microbiome composition (beta diversity) of samples by location. (c) Microbiome variability (beta dispersion) of distal samples among treatments. (d) Microbiome alpha diversity of distal samples among treatments. **Fig. S12:** (a) Sampling schematic of locations (using non-rarefied data) from natural coral colonies that were subjected to snail feeding used for analyses (one-factor test, factor: treatment). (b) Microbiome composition (beta diversity) of samples by location. Letters in legend denote significant differences (*p* < 0.01). (c) Microbiome variability (beta dispersion) of samples by location. (d) Microbiome alpha diversity of samples by location. Letters in legend denote significant differences (*p* < 0.01). **Fig. S13:** Heat map displaying differentially abundant (log_2_ fold > 2) for those ESVs that were greater than 0.5% of the community in either scar, basal, or distal locations on natural colonies fed on by snails. Most ESVs could be identified to the genus or family level (right). Letters indicate significant groupings (FDR-adjusted *p* < 0.01) via Wald’s test with a parametric fit.

**Additional file 3.** Exact sequence variants (ESVs) differing significantly in proportional abundance between coral scar microbiomes from natural coral colonies versus coral scar microbiomes from outplanted corals. Differentially abundant ESVs were identified by DESeq, with significance assessed by a Wald test. The fold change in abundance (expressed as log2) reflects abundance in natural colonies relative to abundance in outplanted corals. ESV classifications are provided at the highest possible taxonomic resolution.

**Additional file 4.** Exact sequence variants (ESVs) differing significantly in proportional abundance between sampling locations on outplanted corals: scar vs. distal microbiomes (Sheet 1), scar vs. basal microbiomes (Sheet 2), and distal vs. basal microbiomes. Differentially abundant ESVs were identified by DESeq, with significance assessed by a Wald test. The fold change in abundance (expressed as log2) reflects abundance in natural colonies relative to abundance in outplanted corals. ESV classifications are provided at the highest possible taxonomic resolution.

## Data Availability

Datasets used in this study are available online from the BCO-DMO data system (http://bcodmo.org/).
