# Supplementary material for: Parasite-host ecology: the limited impacts of an intimate enemy on host microbiomes
Source: Anim Microbiome. 2020 Nov 16;2:42. doi: 10.1186/s42523-020-00061-5 (PMC7807496; doi:10.1186/s42523-020-00061-5)
Supplement: Supplementary file 2 — Additional file 2 Fig. S1. (a) Coralliophila violacea (red arrows) feeding on natural colonies of Porites cylindrica in the field. (b) C. violacea feeding scars (red arrows) on natural P. cylindrica colonies. (c) Experimental set-up assessing the effects of C. violacea feeding on P. cylindrica growth and survivorship. (d) C. violacea feeding scars (red arrows) at the end of the 24 day experiment. Fig. S2: ESV rarefaction curves for each of the 76 coral samples we evaluated. The vertical line indicates sampling depth (8013 sequences). Fig. S3: (a) Sampling schematic of basal locations (using rarefied data) from corals in our manipulative experiment and natural colonies in the field used for analyses (one-factor test, factor: treatment). (b) Microbiome composition (beta diversity) of basal samples among treatments. (c) Microbiome variability (beta dispersion) of basal samples among treatments. (d) Microbiome alpha diversity of basal samples among treatments. Analyses were performed following rarefaction (subsampling without replacement) to a uniform sequence count of 8013 sequences per sample. Fig. S4: (a) Sampling schematic of basal locations (using non-rarefied data) from corals in our manipulative experiment and natural colonies in the field used for analyses (one-factor test, factor: treatment). (b) Microbiome composition (beta diversity) of basal samples among treatments. (c) Microbiome variability (beta dispersion) of basal samples among treatments. (d) Microbiome alpha diversity of basal samples among treatments. Fig. S5: (a) Sampling schematic of distal locations (using rarefied data) from corals in our manipulative experiment and natural colonies in the field used for analyses (one-factor test, factor: treatment). (b) Microbiome composition (beta diversity) of distal samples among treatments. (c) Microbiome variability (beta dispersion) of distal samples among treatments. (d) Microbiome alpha diversity of distal samples among treatments. Analyses were perform [file 42523_2020_61_MOESM2_ESM.docx]

**Fig. S1.** (a) *Coralliophila violacea* (red arrows) feeding on natural colonies of *Porites cylindrica* in the field. (b) *C. violacea* feeding scars (red arrows) on natural *P. cylindrica* colonies. (c) Experimental set-up assessing the effects of *C*. *violacea* feeding on *P*. *cylindrica* growth and survivorship. (d) *C. violacea* feeding scars (red arrows) at the end of the 24 day experiment.


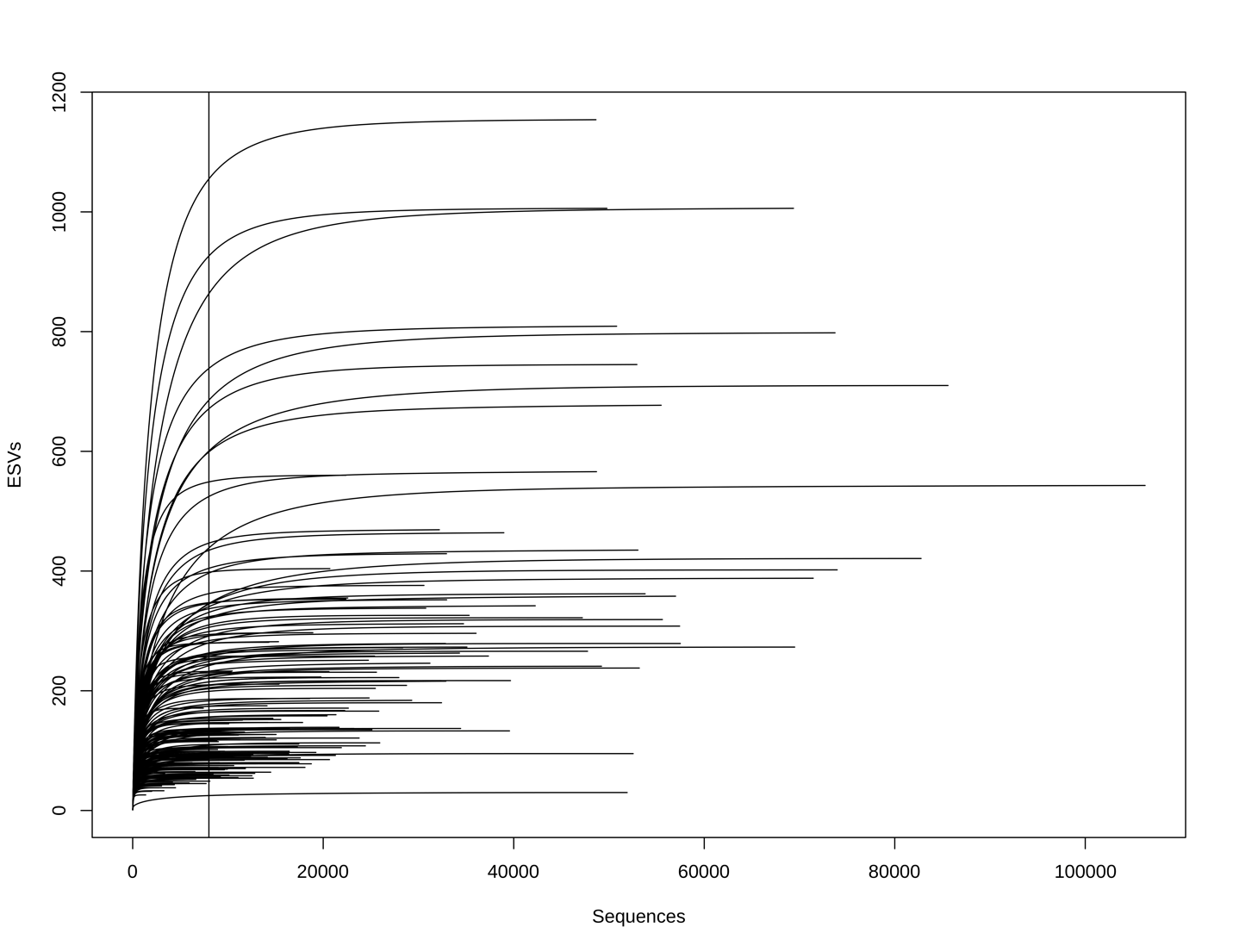


**Fig. S2:** ESV rarefaction curves for each of the 76 coral samples we evaluated. The vertical line indicates sampling depth (8013 sequences).

**Fig. S3:** (a) Sampling schematic of basal locations (using rarefied data) from corals in our manipulative experiment and natural colonies in the field used for analyses (one-factor test, factor: treatment). (b) Microbiome composition (beta diversity) of basal samples among treatments. (c) Microbiome variability (beta dispersion) of basal samples among treatments. (d) Microbiome alpha diversity of basal samples among treatments. Analyses were performed following rarefaction (subsampling without replacement) to a uniform sequence count of 8013 sequences per sample.

**Fig. S4:** (a) Sampling schematic of basal locations (using non-rarefied data) from corals in our manipulative experiment and natural colonies in the field used for analyses (one-factor test, factor: treatment). (b) Microbiome composition (beta diversity) of basal samples among treatments. (c) Microbiome variability (beta dispersion) of basal samples among treatments. (d) Microbiome alpha diversity of basal samples among treatments.

**Fig. S5:** (a) Sampling schematic of distal locations (using rarefied data) from corals in our manipulative experiment and natural colonies in the field used for analyses (one-factor test, factor: treatment). (b) Microbiome composition (beta diversity) of distal samples among treatments. (c) Microbiome variability (beta dispersion) of distal samples among treatments. (d) Microbiome alpha diversity of distal samples among treatments. Analyses were performed following rarefaction (subsampling without replacement) to a uniform sequence count of 8013 sequences per sample.

**Fig. S6:** (a) Sampling schematic of distal locations (using non-rarefied data) from corals in our manipulative experiment and natural colonies in the field used for analyses (one-factor test, factor: treatment). (b) Microbiome composition (beta diversity) of distal samples among treatments. (c) Microbiome variability (beta dispersion) of distal samples among treatments. (d) Microbiome alpha diversity of distal samples among treatments. Analyses were performed using a non-rarefied dataset.

**Fig. S7:** (a) Sampling schematic of scar locations (using non-rarefied data) from corals in our manipulative experiment and natural colonies in the field used for analyses (one-factor test, factor: treatment). (b) Microbiome composition (beta diversity) of distal samples among treatments. (c) Microbiome variability (beta dispersion) of distal samples among treatments. (d) Microbiome alpha diversity of distal samples among treatments.

**Fig. S8:** (a) Sampling schematic of locations (using non-rarefied data) from corals in our manipulative experiment that were subjected to snail feeding used for analyses (one-factor test, factor: location). (b) Microbiome composition (beta diversity) of samples by location. Letters in legend denote significant differences (*p* < 0.01). (c) Microbiome variability (beta dispersion) of samples by location. Letters in legend denote significant differences (*p* < 0.01). (d) Microbiome alpha diversity of samples by location. Letters in legend denote significant differences (*p* < 0.01).


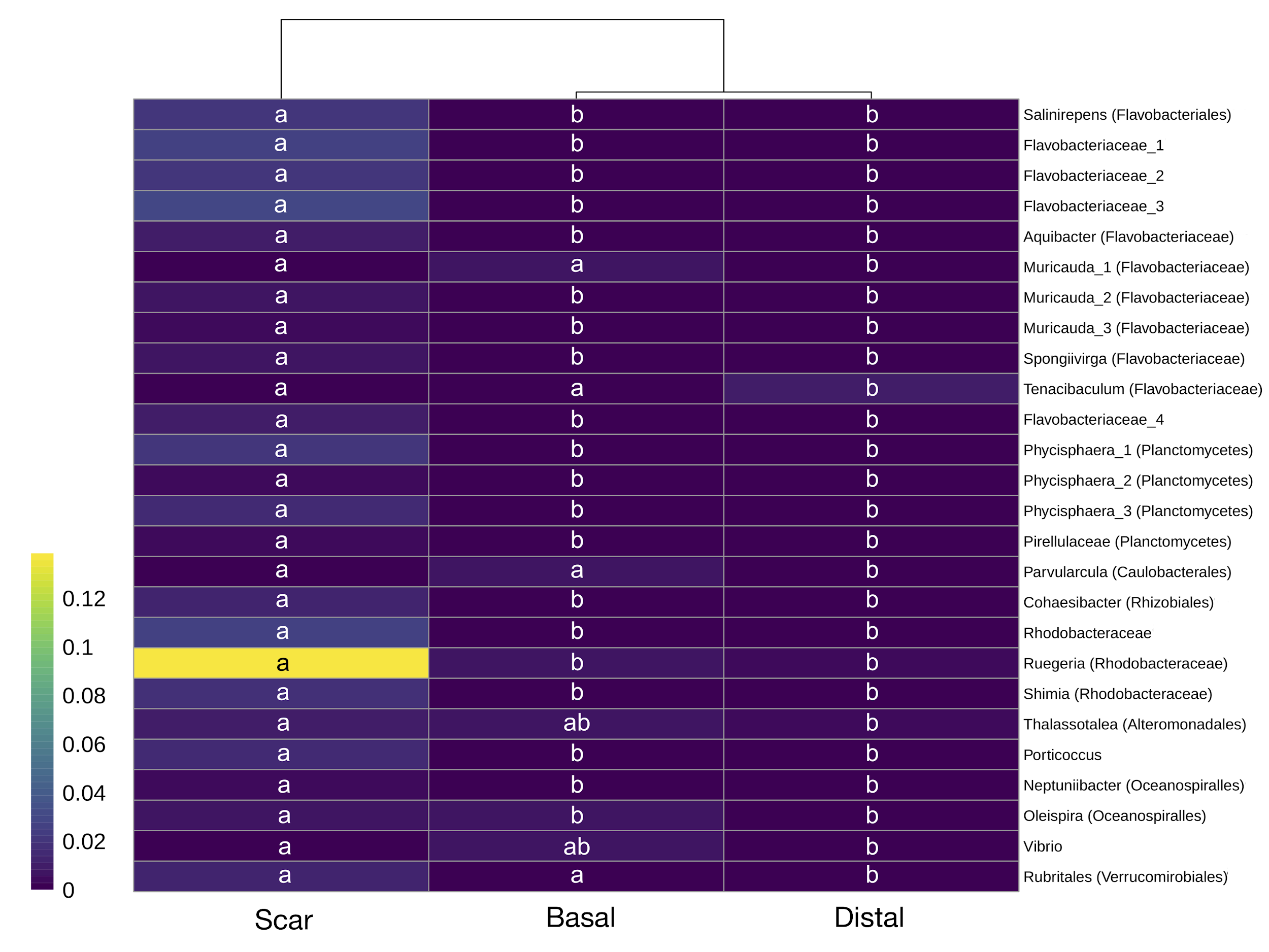


**Fig. S9:** Heat map displaying differentially abundant (log_2_ fold >2) for those ESVs that were greater than 0.5% of the community in either scar, basal, or distal locations on coral outplants fed on by snails. Most ESVs could be identified to the genus or family level (right). Letters indicate significant groupings (FDR-adjusted *p* < 0.01) via Wald’s test with a parametric fit.

**
Fig. S10:** (a) Sampling schematic of basal and distal locations (using rarefied data) from control corals in our manipulative experiment that lacked snails used for analyses (one-factor test, factor: location). (b) Microbiome composition (beta diversity) of samples by location. (c) Microbiome variability (beta dispersion) of distal samples among treatments. (d) Microbiome alpha diversity of distal samples among treatments. Analyses were performed following rarefaction (subsampling without replacement) to a uniform sequence count of 8013 sequences per sample.

**Fig. S11:** (a) Sampling schematic of basal and distal locations (using non-rarefied data) from control corals in our manipulative experiment that lacked snails used for analyses (one-factor test, factor: location). (b) Microbiome composition (beta diversity) of samples by location. (c) Microbiome variability (beta dispersion) of distal samples among treatments. (d) Microbiome alpha diversity of distal samples among treatments.

**Fig. S12:** (a) Sampling schematic of locations (using non-rarefied data) from natural coral colonies that were subjected to snail feeding used for analyses (one-factor test, factor: treatment). (b) Microbiome composition (beta diversity) of samples by location. Letters in legend denote significant differences (*p* < 0.01). (c) Microbiome variability (beta dispersion) of samples by location. (d) Microbiome alpha diversity of samples by location. Letters in legend denote significant differences (*p* < 0.01).


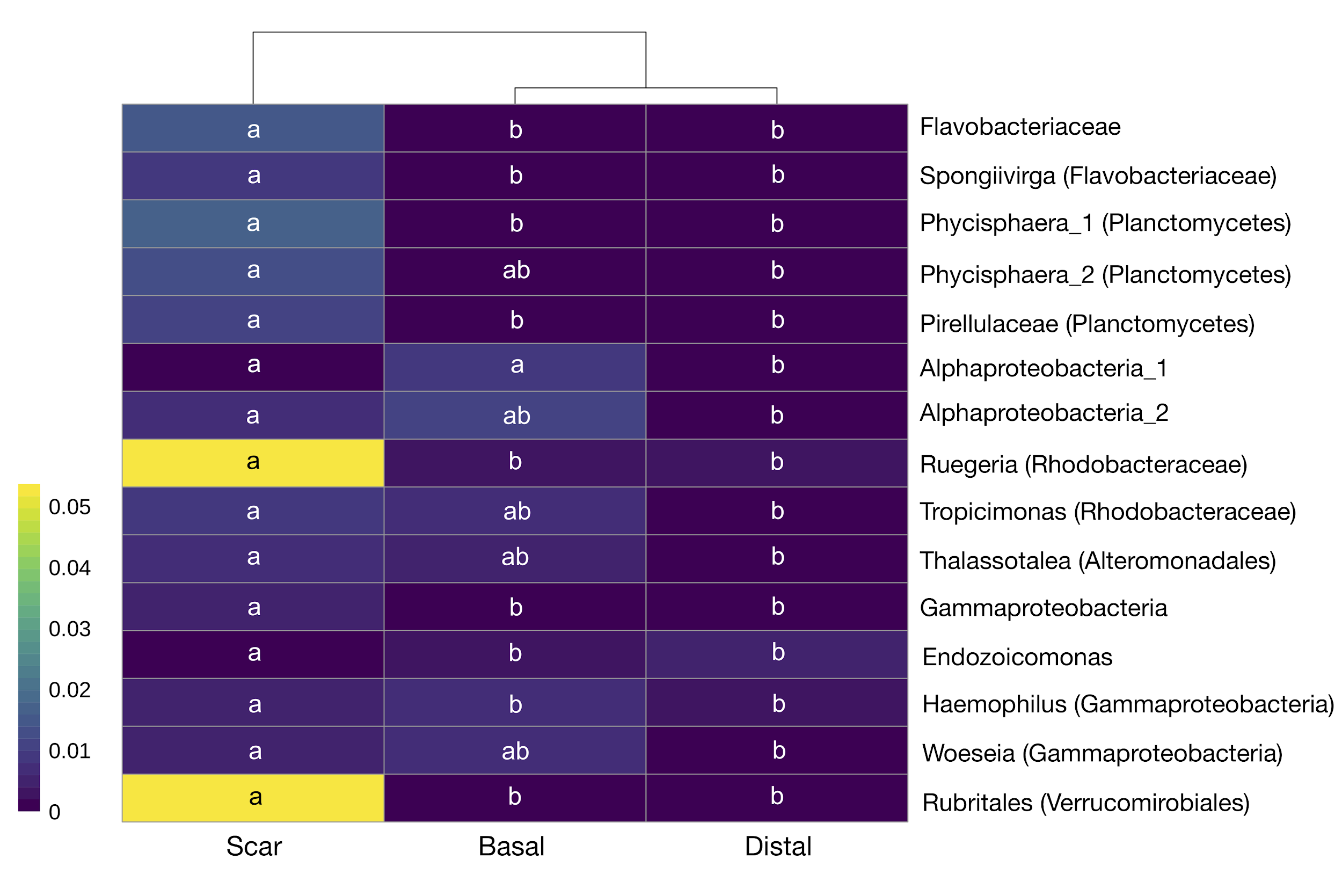


**Fig. S13:** Heat map displaying differentially abundant (log_2_ fold >2) for those ESVs that were greater than 0.5% of the community in either scar, basal, or distal locations on natural colonies fed on by snails. Most ESVs could be identified to the genus or family level (right). Letters indicate significant groupings (FDR-adjusted *p* < 0.01) via Wald’s test with a parametric fit.
